# Embodied Cognition of Manipulative Actions: Subliminal Grasping Semantics Enhance Using-Action Recognition

**DOI:** 10.3390/brainsci15111206

**Published:** 2025-11-08

**Authors:** Yanglan Yu, Qin Huang, Shiying Gao, Anmin Li

**Affiliations:** 1 School of Psychology, Shanghai University of Sport, Shanghai 200438, China; 15295760038@163.com (Y.Y.); gaoshiying68@163.com (S.G.); 2 School of Physical Education, Leshan Normal University, Leshan 614000, China; annnny147@163.com

**Keywords:** embodied cognition, grasping actions, using actions, subliminal semantic processing, event-related potentials (ERPs)

## Abstract

**Background**: Grasping actions, owing to their manipulated nature, play a central role in research on embodied action language. However, their foundational contribution to the cognition of using actions remains debated. This study examined the relationship between grasping and using actions from the perspective of subthreshold semantic processing. **Methods**: Participants engaged with objects affording both action types while behavioral responses and event-related potentials (ERPs) were recorded. Semantic congruency between subliminally presented grasping verbs and the actions of target objects was systematically manipulated. **Results**: Subthreshold processing of grasping verbs facilitated the recognition of using actions, as reflected in faster response times and modulations of ERP components. Spatiotemporal analyses revealed a processing pathway from occipital to parietal and frontal regions, with the posterior parietal cortex serving as a critical hub for integrating object function semantics with action information. **Conclusions**: These findings provide novel evidence that grasping action semantics support the recognition of using actions even below conscious awareness, elucidating the neural dynamics of embodied cognition and refining the temporal characterization of manipulative action processing pathways proposed by the two-action system theory.

## 1. Introduction

Semantic processing refers to the psychological mechanisms by which individuals comprehend and manipulate conceptual information, such as the meanings of words and symbols. It involves the extraction and integration of multimodal inputs—including objects, sounds, faces, and events—and constitutes a core cognitive function closely linked to perception, memory, and thought [1]. Depending on the level of automatization, semantic processing can be classified as either automatic or controlled. With unconscious perception long recognized as a central topic in psychology, research has increasingly demonstrated that subliminal linguistic information can undergo semantic processing [2,3]. For example, semantic priming studies have shown that presenting the word “apple” can activate the associated concept “red” [4], and masked priming experiments have confirmed that prime–target semantic similarity modulates N400 amplitudes even in the absence of conscious awareness [5].

Among these studies, action language has attracted particular attention due to its integration of perceptual, motor, and semantic information [6,7]. Recent accounts of embodied cognition propose that the processing of action language is grounded in sensorimotor systems rather than abstract symbols [6,8], with neural pathways overlapping those involved in action execution and imagery [9]. In other words, the cognitive processing pathways of action language closely resemble those of actual motor processing and are supported by the principles of embodied cognition. Consistent with this view, studies have shown that when verbs directly related to object use are presented as linguistic stimuli, the corresponding motor cortices exhibit significant activation, further demonstrating the embodied nature of action-language processing [10,11]. Within this framework, manipulative actions, owing to their unique operability, have become a primary focus of research on action semantics [12,13]. Human proficiency in tool use has provided a foundation for social progress [14]. Actions directed toward manipulated objects are defined as manipulative actions and are divided into grasping actions [15] and using actions [16] based on their functional goals. Research has shown that presenting object nouns as stimuli enhances memory performance [17]. Moreover, when object nouns are used as primes, participants exhibit significant priming effects when judging target images of hands or feet, accompanied by modulations of early ERP components and the P300 associated with target classification [18]. Similarly, studies have reported that viewing manipulated objects elicits a pronounced parietal P300 component, indicating that such objects capture attentional resources more strongly than non-manipulated ones [19]. Earlier studies further indicate that manipulability influences not only the semantic processing of action information but also serves as a key dimension in the semantic representation of object recognition [20]. Collectively, these findings highlight manipulative actions as a crucial focus within embodied linguistics.

However, the cognitive relationship between the two types of manipulative actions remains a subject of debate. Binkofski and Buxbaum proposed the two-action systems theory, defining the bilateral dorso-dorsal pathway as the structural action system and the left ventro-dorsal pathway as the functional action system. These systems are thought to correspond, respectively, to the neural pathways underlying grasping and using actions [21,22], providing theoretical support for the view that their cognitive processing operates independently. More recent evidence, however, suggests that the recognition of using actions is grounded in the cognitive processing of grasping actions [23,24,25]. For instance, studies have shown that when an object’s grip posture is congruent with its functional use (e.g., holding a knife with the palm forward), reaction times for subsequent using actions (e.g., cutting) are significantly reduced. In contrast, incongruent grip postures (e.g., holding a knife backward) interfere with the initiation of using actions [26,27]. This effect resembles the “spatial alignment effect” observed between an object’s handle orientation and the responding hand [28,29].

Electrophysiological evidence further demonstrates that functional information of manipulated objects modulates hand postures within 200 ms of initiating a grasping action [30], indicating a rapid coupling between semantic processing and motor planning. In addition, research has shown a strong correlation between the processing of action-related linguistic information and ERP component amplitudes, such as the P300 and N400. For example, when participants were asked to judge the type of action associated with target objects, smaller posterior P300 and frontal N400 amplitudes were observed when the priming objects were action-related to the targets. In contrast, primes that were only shape-related but not action-related did not modulate either the P300 or N400 amplitudes [31]. Furthermore, the two-action systems theory emphasizes the critical role of parietal–occipital regions in the neural pathways underlying manipulative action cognition. Whether the processing of action verbs elicits similar ERP components, and whether these components also exhibit activation patterns highlighting the importance of occipital–parietal areas, remains to be clarified. It also remains unclear whether such facilitative effects consistently occur under subliminal semantic processing, and how the temporal dynamics of this relationship unfold. Therefore, the present study, grounded in the framework of embodied linguistics, examined the stability of the facilitative influence of grasping-action semantics on the recognition of using actions at the subliminal level. Moreover, it investigated the temporal course of this effect, providing ERP-based evidence for embodied semantic processing of manipulative actions under subthreshold conditions.

Using the simplicity and transitivity of single-character Chinese verbs, prime stimuli were constructed to represent distinct categories of actions [32]. By varying the semantic congruency between these verbs and the actions of target objects, the study tested whether subliminal grasping-related semantics facilitated subsequent judgments of using actions. Neural responses were assessed with electroencephalography (EEG), focusing on ERP components associated with semantic processing, cognitive control, and action recognition, including the N400 [32,33], P200 [34,35], P600 [36,37], and P300 [31].

## 2. Materials and Methods

### 2.1. Participants

The required sample size was calculated using G*Power 3.1.9.2 version [38]. With an effect size of 0.25, a power of 0.80, and α = 0.05 for a two-factor repeated-measures ANOVA, the minimum required sample was 24 participants. Based on this calculation, 33 undergraduate students were recruited (15 males, 18 females; age range = 19–23 years, mean ± SD = 20.13 ± 1.47 years). Three female participants were excluded due to excessive EEG artifacts, leaving 30 participants (15 males, 15 females) for the final analysis.

All participants were right-handed, had normal or corrected-to-normal vision, a healthy BMI, and no history of neurological, muscular, or psychiatric disorders. None had extensive sports training experience or recent use of psychotropic medication. Written informed consent was obtained after participants were briefed on the study, and they received monetary compensation for their participation. The study was approved by the local ethics committee.

### 2.2. Stimuli

**Figure 1:**
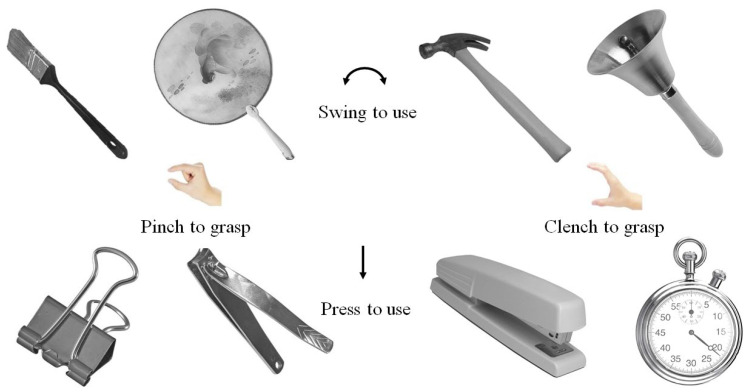
Manipulated object stimuli.

Building on previous research, grasping actions were categorized into pinch and clench types, while using actions were classified as swing and press [39]. These classifications guided the selection of object stimuli. To ensure broad and stable associations between objects and their corresponding action types, 198 participants were recruited to judge and identify the action types associated with candidate objects. Based on these ratings, eight objects were confirmed as experimental stimuli, each paired with a specific combination of grasping and using actions (see Figure 1).

Images of the target objects were drawn from the Bank of Standardized Stimuli (BOSS) [40]. The eight grayscale objects were presented on a 1024 × 768 CRT display at a viewing distance of 45 cm, with a refresh rate of 60 Hz, and were controlled using Psychtoolbox (beta-20190207 V3.0.15) in MATLAB (Matlab2020b.) [41,42]. Each object was displayed at a standardized orientation, with handles tilted 45° to the left, subtending a visual angle of 3.8°. Participants responded via keyboard input, and subsequent event-related potential (ERP) analyses focused on the temporal dynamics of brain activation.

### 2.3. Task and Procedure

The experiment consisted of two phases: an action-type testing phase and the main experimental phase. The action-type testing phase aimed to assess participants’ ability to select the appropriate combination of the two manipulative action types for the target objects, thereby confirming whether participants’ selections could serve as valid prime stimuli in the main semantic priming task. The action-type testing phase lasted approximately 20 min, while the main experimental phase lasted about 60 min.

**Figure 2:**
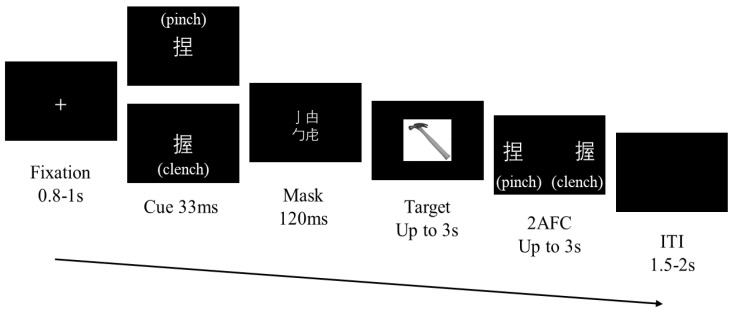
Procedure design for the main experimental phase.

A semantic priming paradigm was employed to investigate the processing of action verbs (Figure 2). Single-character Chinese verbs (‘捏’ for pinch and ‘握’ for clench) served as primes, presented for 33 ms and followed by a 120 ms masking screen. Participants were instructed to classify the using action of target objects (swing or press) as quickly and accurately as possible via key presses. Subsequently, a two-alternative forced-choice (2AFC) task assessed participants’ objective discrimination of the prime stimuli. Trials included randomized inter-stimulus intervals ranging from 1.5 to 2 s. The experiment included four conditions, combining cue–target semantic congruency and using action type, presented across four blocks of 128 trials, with congruent and incongruent trials evenly distributed. Target objects were balanced across the two using-action types.

### 2.4. EEG Data Acquisition

Continuous electroencephalogram (EEG) signals were recorded using the Brain Vision Recorder 2.0 system (Brain Products, Gilching, Germany) with a 64-channel Easy-Cap arranged according to the international 10–20 system. The FCz electrode served as the reference, and AFz as the ground. Vertical electrooculogram (VEOG) recordings were obtained to allow offline correction of eye-movement artifacts. EEG signals were band-pass filtered between 0.01 and 100 Hz and digitized at 500 Hz using a BrainAmp amplifier. Electrode impedances were maintained below 5 kΩ throughout the recording.

### 2.5. EEG Data Analysis

Offline EEG data analysis was performed using the EEGLAB toolbox in MATLAB [43,44]. Independent component analysis (ICA) was applied to attenuate electrooculographic (EOG) artifacts [45]. EEG data were epoched from 200 ms before cue stimulus onset to 2000 ms after target stimulus onset. Trials containing significant artifacts or voltage fluctuations exceeding ±80 µV were excluded. A low-pass filter at 30 Hz was applied, with zeroing at the onset of the target stimulus and baseline correction relative to the 200 ms preceding cue stimulus onset.

Based on previous research and the grand-average ERP waveforms and topographies from the current experiment, four ERP components and regions of interest [46] were defined [46]. For the P200 component [34,35], the time window was 170–220 ms, and the electrodes of interest included P1, Pz, P2, PO3, POz, and PO4; the mean amplitude across these electrodes was calculated. For the P300 component [31], the time window was 280–330 ms, using the same set of electrodes, and the mean amplitude was computed. For the N400 component [47,48], the time window was 320–420 ms, and the electrodes of interest were FC1, FCz, FC2, C1, Cz, and C2; the mean amplitude across these electrodes was calculated. Finally, for the P600 component [36,37], the time window was 480–580 ms, with electrodes P1, Pz, P2, PO3, POz, and PO4; the mean amplitude was computed across these electrodes.

ERP amplitudes were analyzed using repeated-measures ANOVA in SPSS 20.0. Only trials in which participants correctly identified the object’s action type were included; incorrect trials were excluded. Following EEG preprocessing, mean amplitudes were computed for the relevant electrodes under each experimental condition using a 2 (cue–target congruency: congruent vs. incongruent) × 2 (using action type: swing vs. press) design. The number of trials contributing to the ERP analysis in each condition was as follows: 111 for congruent/swing, 109 for congruent/press, 110 for incongruent/swing, and 107 for incongruent/press.

### 2.6. Statistical Methods

To ensure that participants did not have conscious perception of the prime verbs, we employed the four-point Perceptual Awareness Scale (PAS). After each trial, participants reported their awareness of the cue stimulus on a scale from 1 (completely invisible) to 4 (very clear). Trials for which participants reported PAS = 1 were defined as unconscious trials and used for subsequent analyses. In addition, participants’ objective discrimination ability was assessed using one-sample *t*-tests, comparing accuracy, d′, and β values against chance levels (accuracy: 50%; d′: 0; β: 1) to confirm that performance did not exceed chance.

Priming effects were analyzed behaviorally in terms of reaction times and accuracy, and electrophysiologically in terms of mean ERP amplitudes, using a 2 (cue–target semantic congruency: congruent, incongruent) × 2 (using action type: swing, press) repeated-measures ANOVA. Data analysis was conducted in SPSS 20.0. For multiple comparisons, Bonferroni correction was applied. Descriptive statistics are reported as means ± standard errors (SEM). Trials with accuracy below 75% or reaction times exceeding three standard deviations from the mean were excluded.

Mean amplitudes of ERP components for each condition were calculated using scripts in MATLAB. The main statistical analysis focused on predefined ROIs and component-specific time windows. Accordingly, the scalp topographies presented in the manuscript are descriptive visualizations intended to illustrate the distribution of EEG activity under each condition; no statistical comparisons were conducted across conditions for the topographic maps.

## 3. Results

### 3.1. Behavior

Results from the forced-choice task indicated that participants’ accuracy in selecting the priming stimulus text (mean ± SEM = 50.29 ± 0.37%) was comparable to the error rate (mean ± SEM = 49.71 ± 0.40%), both at chance level, with no significant difference between them (paired *t*-test, t(32) = 0.251, *p* = 0.804). This objectively demonstrates that participants were unable to perceive the priming stimuli at the level of visual awareness.

Additionally, we analyzed the discriminability index (d′) and likelihood ratio (β) for participants’ selections of the priming stimuli. The mean ± SEM for d′ was 0.01 ± 0.02, showing no significant deviation from zero (paired *t*-test, t(32) = 0.249, *p* = 0.805), and the mean ± SEM for β was 1.00 ± 0.0002, not significantly different from 1 (paired *t*-test, t(32) = 0.916, *p* = 0.366). These results collectively indicate that participants were entirely unable to discriminate or consciously detect the presence of the priming stimuli (Table 1).

**Table 1:** The results for objective discrimination.

	Mean	SEM	t(32)	*p*
ACC	50.29%	0.37	0.251	0.840
ER	49.71%	0.40		
Dprime	0.01	0.02	0.249	0.850

**Figure 3:**
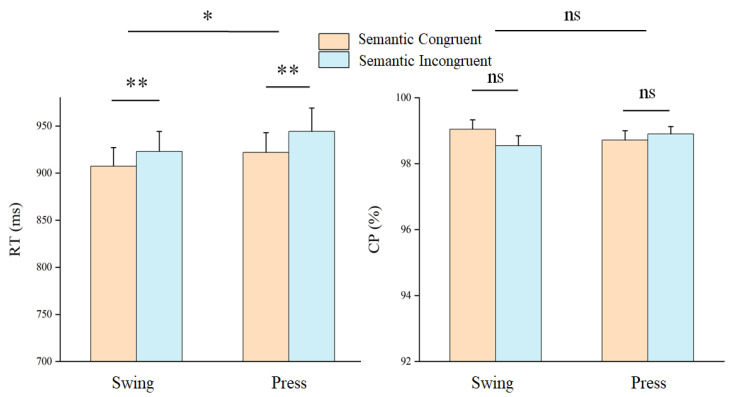
Behavioral results. ns: *p* > 0.05; *: *p* < 0.05; **: *p* < 0.01.

Accuracy and reaction times for object recognition and using actions were analyzed using a two-way repeated-measures ANOVA with the factors semantic congruency (congruent vs. incongruent) and using action type (swing vs. press). Reaction times were significantly faster in congruent trials (congruent: mean ± SEM = 914.60 ± 20.22 ms; incongruent: mean ± SEM = 933.67 ± 22.92 ms, F(1, 32) = 8.480, *p* = 0.006, η^2^p = 0.209). A significant main effect of action type was also observed (F(1, 32) = 6.616, *p* = 0.015, η^2^p = 0.171), with slower responses for press actions compared to swing actions (swing: Mean ± SEM = 933.09 ± 22.69 ms; press: Mean ± SEM = 915.18 ± 20.52 ms). In contrast, accuracy showed no significant effects of semantic congruency (F(1, 32) = 0.575, *p* = 0.454, η^2^p = 0.018) or action type (F(1, 32) = 2.602, *p* = 0.117, η^2^p = 0.075) (Figure 3).

### 3.2. Electrophysiology Components of the Subliminal Priming Task

**Figure 4:**
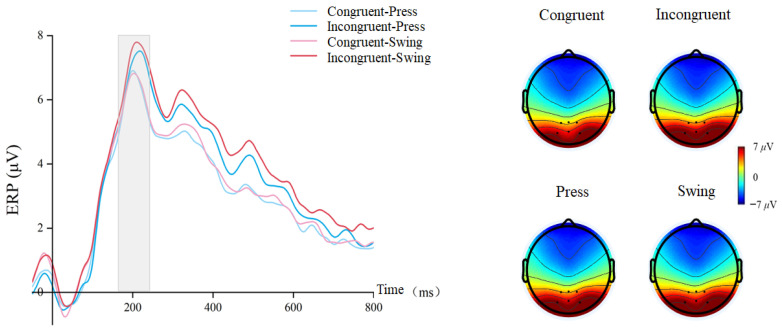
Waveforms and topographical maps for the P200 component.

We conducted a repeated-measures ANOVA on the average amplitude of the P200 component with the factors cue–target stimulus semantic congruency (congruent/incongruent) and using action type (swing/press). This revealed a significant main effect of cue–target stimulus semantic congruency, with smaller amplitudes in congruent trials (congruent: mean ± SEM = 6.421 ± 0.48 μV; incongruent: mean ± SEM = 6.954 ± 0.49 μV, F(1, 29) = 11.969, *p* = 0.002, η^2^p = 0.292) (Figure 4). No significant main effect of action type was observed (F(1, 29) = 3.051, *p* = 0.091, η^2^p = 0.095) and the interaction was not significant (F(1, 29) = 0.066, *p* = 0.799, η^2^p = 0.002).

**Figure 5:**
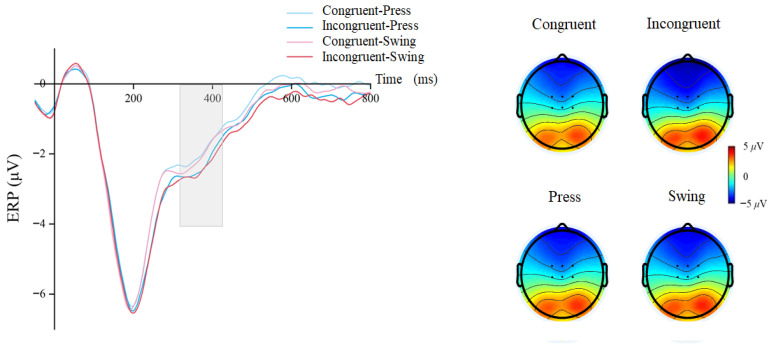
Waveforms and topographical maps for the N400 component.

A repeated-measures ANOVA on the N400 component revealed a significant main effect of cue–target stimulus semantic congruency: incongruent conditions elicited larger N400 amplitudes than congruent conditions (congruent, mean ± SEM = −2.354 ± 0.23 μV; incongruent, mean ± SEM = −2.653 ± 0.26 μV, F(1, 29) = 8.865, *p* = 0.006, η^2^p = 0.234) (Figure 5). No other significant main effects or interactions were observed (using action type, F(1, 29) = 1.088, *p* = 0.306, η^2^p = 0.036; interactions, F(1, 29) = 0.572, *p* = 0.456, η^2^p = 0.019).

**Figure 6:**
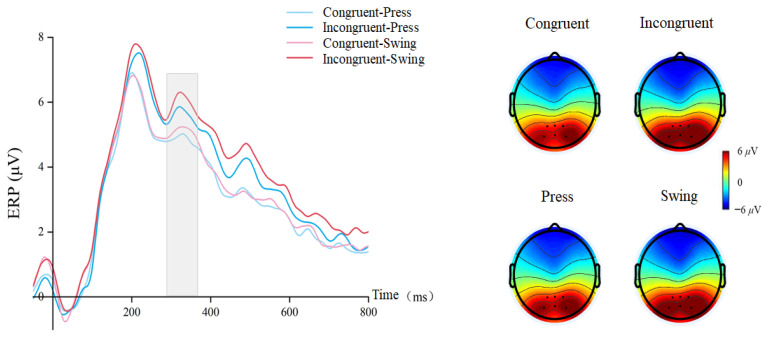
Waveforms and topographical maps for the P300 component.

For the P300 component, a repeated-measures ANOVA revealed a significant main effect of semantic congruency (F(1, 29) = 21.055, *p* < 0.001, η^2^p = 0.421), with smaller amplitudes in congruent conditions (congruent: mean ± SEM = 5.047 ± 0.21 μV; incongruent: mean ± SEM = 5.922 ± 0.29 μV) (Figure 6). There was also a significant main effect of using action type (swing: mean ± SEM = 5.652 ± 0.25 μV; press: mean ± SEM = 5.317 ± 0.24 μV, F(1, 29) = 15.425, *p* < 0.001, η^2^p = 0.347). No significant interaction was observed (F(1, 29) = 1.062, *p* = 0.311, η^2^p = 0.035).

**Figure 7:**
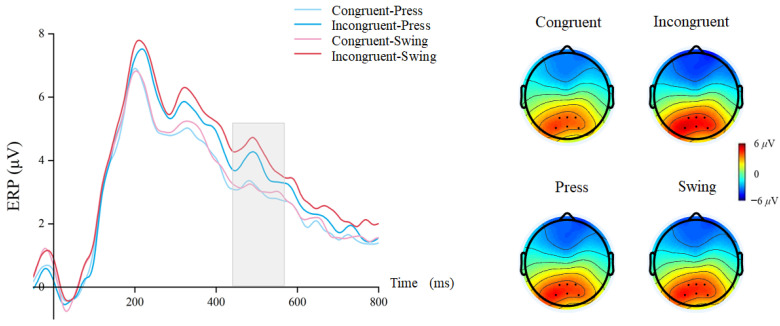
Waveforms and topographical maps for the P600 component.

Finally, for the P600 component, repeated-measures ANOVA revealed a significant main effect of cue–target stimulus semantic congruency: incongruent conditions elicited larger amplitudes than congruent conditions (congruent, mean ± SEM = 3.045 ± 0.25 μV; incongruent, mean ± SEM = 4.016 ± 0.29 μV, F(1, 29) = 30.588, *p* < 0.001, η^2^p = 0.513) (Figure 7). Similar to the P300, there was a significant main effect of action type (swing: mean ± SEM = 3.658 ± 0.26 μV; press: mean ± SEM = 3.404 ± 0.27 μV, F(1, 29) = 6.207, *p* = 0.019, η^2^p = 0.176), but no significant interaction was observed (F(1, 29) = 4.025, *p* = 0.057, η^2^p = 0.121).

## 4. Discussion

In this study, we introduced the concept of semantic processing of manipulative actions within the framework of embodied cognition in action language, specifically examining whether action verbs exhibit an embodied cognitive representation. In other words, we investigated whether the neural pathways for manipulative action recognition proposed by the two-action system theory also show overlapping activation patterns during the subthreshold semantic processing of these verbs. Object stimuli were categorized according to different types of manipulative actions, allowing us to identify a facilitatory effect of subthreshold semantic processing of grasping actions on the recognition of using actions. This finding provides empirical support at the subthreshold semantic processing level for the hypothesis that grasping actions constitute a cognitive foundation for using actions. Moreover, event-related potential (ERP) techniques were employed to examine the temporal dynamics of this semantic facilitation effect.

To ensure unconscious processing of the prime words, we conducted an objective discriminability test. Participants’ accuracy in the forced-choice task was 50.29%, and the discriminability index (d′) did not differ from zero, indicating no objective discriminative ability. These results demonstrate that participants’ selection of the prime words remained at chance level, confirming that the primes were presented below the threshold of visual awareness and providing a solid methodological basis for investigating subthreshold semantic processing of actions.

Behavioral data analysis revealed that response times in the using-action judgment task were faster when the prime and target stimuli shared semantically congruent grasping actions compared to incongruent conditions, indicating a positive priming effect of semantic congruency on the recognition of using actions. Additionally, differences in the cognitive processing of the two types of using actions—“press” and “swing”—contributed to variations in response speed, with responses for the press action being significantly faster than those for the swing action.

A similar action-type effect was observed in the amplitudes of the P300 and P600 components. The P300 component, associated with the allocation of cognitive resources and task difficulty, was significantly modulated by the linguistic information of manipulative actions. By contrast, the posterior P600 component has been linked to semantic conflict or conflict resolution [49]. These results indicate that the cognitive complexity of the two using-action types differed, leading to variations in the amplitudes elicited by each action type [50].

Furthermore, these findings are consistent with the embodied processing framework of action language, which posits that the processing of using-action representations is tightly coupled with the body and motor systems and exhibits contextual dependence [51]. For example, whether an action is performed toward or away from the body creates context-dependent differences in action processing [7]. Embodied semantics further suggests that the semantic activation of action-related words is mapped onto bodily schemata, resulting in differential representations for distinct action types within the sensorimotor system [52]. In the present experiment, swing actions—relative to press actions—represented movements directed away from the body with less contextual guidance, rendering their recognition more complex. From the perspective of embodied cognition, which emphasizes shared neural resources between lexical and action processing, this increased complexity likely required greater cognitive resources. These findings further support previous research highlighting the critical role of the parieto-occipital regions in the recognition of manipulative actions [53,54].

In addition, a main effect of semantic congruency between prime and target stimuli was observed across the P200, N400, P600, and P300 components. The N400 amplitude was significantly smaller in semantically congruent conditions compared to incongruent conditions, demonstrating that even unconscious grasping verbs can undergo successful semantic processing [55,56]. A similar pattern emerged for the P600 component, indicating that comprehension of semantic conflict under incongruent conditions requires greater resource allocation [49]. Likewise, the P300 amplitude was modulated by semantic congruency, suggesting that participants processed grasping-action information, specifically grasping verbs [31,57], which in turn facilitated the recognition of using actions [58]. Collectively, these findings indicate that semantic processing of grasping manipulative actions exerts a facilitatory effect on the cognition of using manipulative actions, even at a subthreshold level. This confirms the stability of the facilitation effect and provides further empirical support for the hypothesis that grasping actions constitute a cognitive foundation for using actions [25,26,59].

Moreover, the ERP results revealed the cognitive pathways underlying semantic processing of manipulative actions. Differences in semantic congruency first emerged in occipital regions associated with visual feature processing, then progressed to the posterior parietal cortex for decision-related updates, followed by frontal regions for deeper semantic integration, and finally reached parieto-occipital regions implicated in higher-order semantic conflict recognition. This temporal sequence aligns closely with the processing pathways proposed by the two-action system theory, emphasizing the critical role of the parieto-occipital regions and the integrative function of frontal areas [21,22]. Accordingly, our study provides a temporal-level refinement and extension of the two-action system’s proposed cognitive pathways for manipulative actions within the framework of action language processing.

Although the observed facilitation effects could also be explained by symbolic semantic priming mechanisms, the action-specific nature of the congruency modulation—particularly between grasping and using actions—suggests an underlying sensorimotor resonance consistent with embodied cognition [17,60]. It is important to note that this study only used action-related verbs as stimuli. Future research could incorporate direct sensorimotor tasks as baseline conditions to more comprehensively explore the embodied neural mechanisms underlying manipulative action semantics. The single-character Chinese verbs used, characterized by transitivity and structural simplicity, allowed precise assessment of manipulative action semantic processing [61]. The selected verbs had highly consistent structural composition, ensuring that observed effects were driven by action-specific semantics rather than structural variability. Nevertheless, the current stimuli have limitations, and future studies could include non-action primes to further dissociate action-specific from general semantic effects. It is also important to recognize that ERP components such as the P200, N400, and P600 do not index single cognitive processes in isolation. Rather, they likely reflect overlapping neural activities across distributed cortical networks. Accordingly, the observed occipital-to-frontal topographic progression should be interpreted as a temporal sequence of scalp-level activations reflecting stages of visual, semantic, and cognitive integration, rather than direct source localization. Future studies employing source reconstruction or multimodal imaging (e.g., EEG–fMRI) could help clarify the neural generators underlying these temporal dynamics.

In summary, our findings demonstrate that semantic processing of grasping actions effectively facilitates the recognition of using actions. Significant differences were also observed between different types of using actions: specifically, press actions were recognized more quickly than swing actions, with corresponding neural activity in parietal and occipital regions associated with contextual updating and fine-grained discriminative processing [53,54]. The subthreshold semantic priming effect of grasping actions on using actions was reflected behaviorally in faster action recognition and neurally in facilitative changes within dorsal brain regions. Building on the neural pathways for manipulative action recognition proposed by the two-action system theory, this study examined the temporal dynamics of subthreshold semantic processing of manipulative actions, thereby refining and extending the two-action system framework [21,22]. These findings provide indirect evidence for the embodied nature of subthreshold semantic processing of manipulative actions and carry practical significance. By highlighting the foundational role of grasping information in cognition and the importance of parietal–occipital integration, this study offers a bioinspired blueprint for developing more intuitive human–machine interfaces and robotic grasping strategies. For example, designing graspable regions of intelligent tools (corresponding to grasping actions) to naturally guide correct usage (corresponding to using actions) could enhance interaction fluency. Moreover, for patients with apraxia resulting from brain injury (e.g., stroke), difficulties often arise from an inability to translate object knowledge into appropriate actions. The ERP paradigm established here may serve as a sensitive diagnostic tool to distinguish whether deficits occur at the level of action semantics or during action selection and integration [62]. Based on this, targeted rehabilitation interventions—such as subliminal priming techniques—could subtly rebuild and strengthen impaired action–semantic pathways.

## 5. Conclusions

Guided by the framework of embodied linguistics, the present study demonstrated that subthreshold semantic processing of grasping actions effectively facilitates the recognition of using actions. Behavioral and ERP results jointly indicate that grasping verbs constitute a robust cognitive foundation for identifying using actions. Spatiotemporal analyses of ERP data revealed a processing pathway from occipital to parietal and then frontal regions, with the posterior parietal cortex serving as a central hub for integrating object function semantics with action information. These findings not only extend the temporal characterization of manipulative action processing pathways proposed by the two-action system theory but also provide empirical support for the embodied nature of action language processing at a subthreshold level. Overall, the study underscores the pivotal role of grasping action semantics in shaping action recognition and supports the embodied cognition framework for action language from the perspective of subthreshold manipulative action processing.

## Data Availability

The original contributions presented in this study are included in the article. Further inquiries can be directed to the corresponding author.
